# Data on empirical estimation of the relationship between agency costs and ownership structure in Italian listed companies (2002–2013)

**DOI:** 10.1016/j.dib.2018.04.106

**Published:** 2018-05-03

**Authors:** Fabrizio Rossi, James R. Barth, Richard J. Cebula

**Affiliations:** a Economics and Business Organisation, University of Cassino and Southern Lazio, via G. Di Biasio 43 Cassino, FR 03043, Italy; b Lowder Eminent Scholar in Finance at Auburn University, AL, USA; c Walker/Wells Fargo Endowed Chair in Finance at Jacksonville University, FL 32211, USA

**Keywords:** G32, G34, Agency theory, Debt, Ownership structure, Multiple blockholders, Family-controlled firms, Generalized method of moments (GMM) estimation

## Abstract

The data presented in this article are related to the research article entitled “Do shareholder coalitions affect agency costs? Evidence from Italian-listed companies”, *Research in International Business and Finance*, Forthcoming (Rossi et al., 2018) [1]. The study shows an empirical analysis using an extensive balanced panel dataset of 163 Italian listed companies for the period 2002–2013, which is a sample yielding 1956 firm-year observations. The sample consists primarily of manufacturing firms, but also includes some service enterprises. However all financial firms and regulated utilities are excluded. We collected data on ownership structure for the entire study period. Information was acquired from the Consob website and the individual company reports on corporate governance. Data on firm-level indicators (debt-to-capital ratio, firm size, and age of the firm) for all companies in the sample were collected from *Datastream, Bloomberg*, and *Calepino dell’Azionista*, as well as obtained manually from the financial statements of the individual companies being studied. Our dataset contains several measures of ownership structure for Italian listed companies.

**Specifications table**

**Table:** 

Subject area	*Corporate finance, corporate governance*
More specific subject area	*Agency costs, ownership structure, coalitions among non-controlling shareholders, Panel data, Generalized Method of Moments (GMM) estimation*
Type of data	*Raw data*
How data was acquired	*Manually from reports on corporate governance, financial statements, Calepino dell’Azionista, CONSOB, and downloaded by Datastream and Bloomberg*
Data format	*Raw data*
Data source location	*Italy*
Data accessibility	*The raw data are on our personal dataset. Below is the link where the reader can find the data on ownership structure for Italian listed companies*
	http://www.consob.it/web/consob-and-its-activities/listed-companies
Related research article	Rossi et al. (2018) [1]

**Value of the data**

• Our dataset used in Rossi et al. [1] contains several measures of ownership structure for Italian listed companies.
• The dataset provides firm-level indicators used in the sample for the period 2002–2013.
• The dataset also allows one to examine the descriptive statistics of the firm-level indicators.
• Our dataset can be used for further studies on Italian listed companies and can be used for a comparative analysis with other common law and civil law countries, such as Spain, France, Germany, and the UK.

## 1 . Data

The data provided and explained here (Table 1) refer to independent and dependent variables used in our research. We allow a reader to see data on the three largest shareholder (*OWNCONC*), total debt scaled by total assets (*DEBT*), a proxy of agency costs (*Sales-to-asset ratio*), the share held by the board of directors (*BOWN*), firm age (*LOG AGE*), the size of the firms (*LOG SIZE*), and the shareholder type (*Family firms*). We calculate family-controlled firms using a dummy variable that takes a value equal to 1 if the firm is family controlled and a value 0 if the firm is not family controlled. We also show in Table 1 the methodology used to define the family controlled firm.

**Table 1:** Definitions and source of variables used in our analysis.

Variables	Definition	Source	Column
*Dependent Variable*			
SALES-TO-ASSET RATIO	Net sales scaled by total assets (adjusted for median industry)	Our calculation by Datastream, Blomberg, *Calepino dell’Azionista* (Mediobanca), and Financial statements of the individual companies	(7)
*Independent Variables*			
DEBT	Total debt scaled by total assets	Our calculation by Datastream, Blomberg, *Calepino dell’Azionista* (Mediobanca), and Financial statements of the individual companies	(6)
SIZE (LOG SIZE)	Log of total sales	Our calculation by Datastream, Blomberg and *Calepino dell’Azionista* (Mediobanca), and Financial statements of the individual companies	(3)
FIRMAGE (LOG AGE)	Years by firm establishment (in logarithmic form)	Our calculation by *Calepino dell’Azionista* (Mediobanca) [3]	(9)
OWNCONC	The sum of the three largest shareholders	Our calculation by *Commissione Nazionale per le Società e la Borsa* (Consob) [2]	(4)
FAMILY	Dummy=1 if there is an individual shareholder at 20% of stake; 0 otherwise	Our calculation by *Commissione Nazionale per le Società e la Borsa* (Consob)	(8)
BOWN	The percentage of shares owned by the board of directors	*Commissione Nazionale per le Società e la Borsa* (Consob) and Corporate Governance reports of firms.	(5)
FIRMS	The number of firms of our sample	We exclude all financial firms (SIC code 6000-6999) and regulated utilities (SIC code from 4900-4999)	(1)
YEAR	The number of years for each firm involved in our sample	We consider the period 2002–2013 for 1956 firm-year observations	(2)

Moreover, our data show in the first and second columns the number of firms used in our sample (*Firms*) and the years investigated (*Year*) for each firm, respectively.

## 2 Methodology

The study adopts a dynamic panel data model involving a two-step system-GMM (Generalized Method of Moments) because it is a powerful econometric tool that captures the two components of endogeneity, namely, that which is attributable to unobservable heterogeneity and that which is associated with simultaneity. It addresses endogeneity problems through the use of a set of lagged explanatory variables as instruments for the explanatory variables.
